# Content of Heavy Metals in Animal Feeds and Manures from Farms of Different Scales in Northeast China

**DOI:** 10.3390/ijerph9082658

**Published:** 2012-07-27

**Authors:** Fengsong Zhang, Yanxia Li, Ming Yang, Wei Li

**Affiliations:** 1 State Key Laboratory of Environmental Criteria and Risk Assessment, Chinese Research Academy of Environmental Sciences, Beijing 100012, China; Email: zhangfs@craes.org.cn; 2 State Key Laboratory of Water Environment Simulation, School of Environment, Beijing Normal University, Beijing 100875, China; Email: Yangming_shida@163.com (M.Y.); gscas2012@163.com (W.L.)

**Keywords:** heavy metal, livestock feed, animal manure, Northeast China

## Abstract

To determine the contents of heavy metal (Cu, Zn, As, Cr, Cd and Pb) in animal feeds and manures, 104 livestock feeds and 118 animal manure samples from farms of different herd size and located in northeast China were collected and their heavy metal concentrations were determined. The content of Cu, As and Cd ranged from 2.3–1,137.1 mg/kg dm, 0.02–13.03 mg/kg dm and non-detectable (nd)−31.65 mg/kg dm in pig feeds, 2.88–98.08 mg Cu/kg dm, 0.02–6.42 mg As/kg dm and non-detectable (nd)–8.00 mg Cd/kg dm in poultry feeds, and their content in cattle feeds was similar to that in poultry feeds. The typical content in pig manures was 642.1 mg Cu/kg dm, 8.6 mg As/kg dm, and 15.1 mg Cd/kg dm, which reflected the metal contents in feeds. The typical contents in poultry manures were 65.6 mg Cu/kg dm, 3.3 mg As/kg dm and 1.6 mg Cd/kg dm while the contents in cattle manures were 31.1 mg Cu/kg dm, 2.5 mg As/kg dm and 0.5 mg Cd/kg dm. Animal manure is an important source of heavy metals to the environment in Northeast China.

## 1. Introduction

Due to the rapid development of intensive animal farms in China, animal manure production is about 32 × 10^8^ t annually [1]. Commonly, animal manure was applied to agricultural land to improve the soil fertility and organic matter content. However, this practice also results in serious environmental problems, such as nitrate and phosphate contamination of surface waters [2]. Another important problem induced by animal manure application is metal pollution, as animal manure contains high metal (Cu, Zn, As, Cd) concentrations [3,4,5]. Residues of heavy metals in manures can be accumulated in surface soils as a result of long-term agricultural use [6,7,8]. Accumulation of heavy metals could not only affect the soil fertility and the product quality [9], but also promote metal migration through leaching and runoff [10,11]. Therefore, due to potential risks of heavy metal pollution heavy metal residues in animal manure have received scientific attention [12,13].

A wide range of heavy metals in animal manures has been investigated in intensive animal production in China [14,15,16,17]. According to the report of Li *et al*., the content range of Cd in pig manures ranged from non-detectable (nd) to 129.76 mg/kg dry matter in Beijing and Fuxin [18]. Furthermore, heavy metal residue displayed diversity in content in different provinces of China. For example, average Cu content in pig, cattle and chicken manures from Jiangsu province was 399, 46 and 89 mg/kg dry matter [14], which were similar to those in England and Wales reported by Nicholson *et al*. [19]. However, pig and chicken manure contained higher values of 765 and 107 mg/kg dry matter from Guangdong province, which is a developed livestock husbandry area in China [20]. In the same area, heavy metal residues in animal manures also showed diversity in farms of different scales. Super-intensive farms displayed higher Cu concentration than middle scale farms [16]. 

It has been recently reported that the content of Cu, As and Cd in farmlands was increased in the northeast of China, which was an important grain-production region [21]. Luo *et al*. [13] confirmed that animal manure was an important source of soil pollution caused by heavy metals in China. Xiong *et al*. [17] suggested that the application of manure would enhance the risk of Cu contamination in Fuxin city in Northeast China. In recent decades, the economic boom has stimulated the demand for animal products and consequently led to a vast expansion of animal production. Approximately 9.25 × 10^8^ pigs, 2.54 × 10^8^ cattle and 1.58 × 10^9^ chickens were raised in Northeast China annually [22]. It is a common practice to add minerals such as copper (Cu), zinc (Zn) and arsenic (As) to animal feeds via mineral additives because of their antimicrobial and growth-stimulating effects [16,19]. However, so far, the impacts of feed additives application and heavy metals residue in animal manures in the northeast of China remain unknown. In this study, for the convenience of constructing more accurate metal budgets and calculating the average rates of heavy metal inputs in agricultural land, we sampled animal feeds and manures from animal farms of different scales to determine the contents of heavy metals in this area. 

## 2. Materials and Methods

### 2.1. Sampling and Analysis

Northeast China (38°43′–53°33′N, 118°53′–135°05′E) is a geographical region of China consisting of three provinces: Liaoning, Jilin and Heilongjiang. Animal manures and feeds were randomly sampled from farms of different scales in the three northeastern provinces. All farms investigated in this survey were classified into three groups according to their animal population (Table 1). In all, 222 poultry and livestock samples, including 104 feed samples and 118 manure samples were collected. 

**Table 1:** Animal population in farms of different scales.

Farm scale	Animal population		
	Pigs	Chickens	Cattle
Small	<200	<2,000	<100
Middle	200–800	2,000–20,000	100–300
Large	>800	>20,000	>300

Fresh feed and manure samples were air-dried in the shade, then ground and passed through a 0.25-mm mesh PVC sieve. Subsamples of 0.5 g was collected from the dry powder and then digested in heated, concentrated HNO_3_ and H_2_O_2_ [23]. The content of Cu, Zn, Cr, Pb and Cd in the filtered supernatant was determined by graphite furnace atomic absorption spectrometry (AAS, Vario6, Jena Co. Ltd., Jena, Germany), and As in filtrates was analyzed with a hydrogen generation atomic fluorescence spectroscope. The accuracy of analysis was checked using wheat and soil samples with certified contents (GSS-1 and GBW-08501 respectively, China National Center for Standard Materials). The recovery of heavy metal ranged from 97.5% to 101.1% in GSS-1 and GBW-08501. A 15% parallel replication of samples was also used as a quality control procedure. 

### 2.2. Statistical Analysis

Normality and homogeneity of the variances were checked using the Shapiro-Wilk and Levene tests before ANOVA analysis, respectively. Due to the wide range of variation, normalization was developed using log_10_ function before ANOVA analysis in some parameters. For the data not conforming to normal distribution after data transformation, non-parametric method was applied in some statistical tests. SPSS 13.0 statistical package for Windows was used for data analysis. 

## 3. Results

### 3.1. Contents of Heavy Metals in Animal Feeds

Results of the feed sample analyses on a dry matter (dm) basis from farms of different herd size are listed by livestock type in Table 2, Table 3, Table 4. Contents of Pb are not reported here as its values were lower than the detection limit in all feed samples. All feed samples contained Cu, Zn and As, indicating that these additives were widely applied in animal production in Northeast China. Cr in cattle feeds was almost below the detection limit; while Cr was detected in over 13% of pig feeds and 20% of chicken feeds. Although Cd is not necessary for animal growth, over 60.6% of feed samples were detected to contain this toxic metal in the survey. 

**Table 2:** Heavy metals in cattle feed from farms of different herd size (mg/kg dm).

Herd size	No. of farms	Metals	Cu ^1^	Zn ^1^	As ^2^	Cr ^2^	Cd ^2^
<100	11	Range	2.73–114.68	24.62–346.12	0.05–2.81	nd–0.05	nd–23.25
		Mean	32.12 ^a^	156.28 ^a^	1.38 ^a^	nd ^a^	2.31 ^a^
		Median	19.08	119.32	1.49	nd	0.30
100–300	11	Range	2.73–61.48	26.27–215.92	0.44–2.42	nd	nd–1.00
		Mean	15.74 ^a^	101.78 ^a^	1.18 ^a^	nd^a^	0.38 ^a^
		Median	8.53	112.62	1.28	nd	0.35
>300	17	Range	4.58–113.14	11.07–334.86	0.01–6.12	nd	nd-13.23
		Mean	25.98 ^a^	114.90 ^a^	0.80 ^b^	nd ^a^	2.03 ^a^
		Median	13.63	91.00	0.41	nd	0.03

nd: non-detectable. Different letters (superscripts) in the same column indicate the significant difference between the mean contents of different farms (*p* < 0.05) by ^1^ Tukey test and ^2^ Kruskal-Wallis H-test.

**Table 3:** Heavy metals in chicken feed from farms of different herd size (mg/kg dm).

Herd size	No. of farms	Metals	Cu ^1^	Zn ^1^	As ^1^	Cr ^2^	Cd ^2^
<2,000	6	Range	2.88–10.28	52.62–111.12	0.08–1.91	nd–39.80	nd
		Mean	6.28 ^a^	74.91 ^b^	0.59 ^a^	6.63 ^a^	nd ^a^
		Median	5.93	72.62	0.12	nd	nd
2,000–20,000	14	Range	2.88–51.73	63.12–127.32	0.04–3.36	nd–936.45	nd–1.70
		Mean	16.75 ^a^	89.34 ^ab^	0.75 ^a^	66.96 ^a^	0.26 ^a^
		Median	10.73	81.35	0.49	nd	0.13
>20,000	10	Range	4.47–98.08	74.07–150.97	0.02–6.42	nd–160.25	nd–8.00
		Mean	21.87 ^a^	103.72 ^a^	1.22 ^a^	25.72 ^a^	1.03 ^a^
		Median	13.43	104.46	0.68	nd	0.16

nd: non-detectable. Different letters (superscripts) in the same column indicate the significant difference between the mean contents of different farms (*p* < 0.05) by ^1^ Tukey test and ^2^ Kruskal-Wallis H-test.

**Table 4:** Heavy metals in pig feeds from farms of different herd size (mg/kg dm).

Herd size	No. of farms	Metals	Cu ^2^	Zn ^1^	As ^1^	Cr ^2^	Cd ^2^
<200	7	Range	32.33–1,137.07	57.27–598.32	0.04–13.03	nd–1.35	nd–31.65
		Mean	343.38 ^a^	238.91 ^a^	5.62 ^a^	0.23 ^a^	8.73 ^a^
		Median	107.28	122.42	1.60	nd	1.02
200–800	7	Range	10.78–164.08	38.92–128.12	0.07–2.68	nd–11.7	nd–0.35
		Mean	93.92 ^a^	88.00 ^a^	1.00 ^a^	1.62 ^a^	0.18 ^a^
		Median	90.43	95.92	0.65	nd	0.25
>800	21	Range	2.33–258.08	37.37–206.72	0.02–10.95	nd	nd–27.60
		Mean	117.70 ^a^	135.88 ^a^	2.22 ^a^	nd ^a^	3.70 ^a^
		Median	119.88	138.37	1.08	nd	0.04

nd: non-detectable. Different letters (superscripts) in the same column indicate the significant difference between the mean contents of different farms (*p* < 0.05) by ^1^ Tukey test and ^2^ Kruskal-Wallis H-test.

The contents of Cu, Zn, As and Cd in all cattle feed samples were in the ranges of 2.73–114.68 mg/kg, 11.07–346.12 mg/kg, 0.01–6.123 mg/kg and non-detectable (nd)–23.25 mg/kg. The metals of highest contents in cattle feeds from large farms were Zn and Cu, with mean contents of 114.90 mg/kg and 25.98 mg/kg, respectively. No significant difference was observed in the average contents of same heavy metal in cattle feeds from farms of different herd size except for As. In this survey, more As was found in cattle feeds from small farms than middle and large farms. 

Cr was one of the most variable elements in all chicken feed samples, with a wide range between non-detectable (nd) to 936.45 mg/kg. Contents of Cd and As were <8 mg/kg in all the compound feeds analyzed. Contents of Cu in chicken feeds were typically in the range of 2.88–98.08 mg/kg, whilst Zn contents ranged from 52.62 to 150.97 mg/kg. In terms to the mean content, chicken feeds from large farms contained significantly higher Zn than that from small farms (*p* < 0.05, Tukey test). 

The contents of Cu, Zn, As and Cd in all pig feed samples covered a wide range, 2.3–1,137.1 mg/kg, 37.37–598.32 mg/kg, 0.02–13.03 mg/kg and non-detectable (nd)–31.65 mg/kg. Like cattle feeds, the metals of highest contents in pig feeds were also Zn and Cu. With respect to average content, pig feeds contained higher Cu than cattle and chicken feeds. There was no significant difference in the average contents of the same heavy metal in pig feeds from farms of different herd size. However, the mean Cu in pig feeds from small farms was three and four times higher than those from middle and large farms, respectively. 

### 3.2. Contents of Heavy Metals in Animal Manures

The contents of six heavy metals (Cu, Zn, As, Cr, Cd, Pb) in manures from different farms are shown in Table 5, Table 6, Table 7. In cattle manures, the contents of Cu, Zn and As were 10.28–112.90 mg/kg, 16.97–377.17 mg/kg and 0.46–19.44 mg/kg, respectively. The contents of Cr, Cd and Pb in cattle manures were <11 mg/kg. The average contents of Cu, Zn, As, Cr, Cd and Pb in large farms were 31.37, 136.13, 2.71, 0.24, 0.73 and 2.68 mg/kg. Like the feeds, the metal of highest contents in cattle manure was Zn and Cu. Apart from Cd, there was no significant differences in the contents of heavy metal observed from farms of different herd size. 

**Table 5:** Heavy metals in cattle manure from farms of different herd size (mg/kg dm).

Herd size	No. of farms	Metals	Cu ^1^	Zn ^1^	As ^1^	Cr ^2^	Cd ^2^	Pb ^1^
<100	13	Range	14.08–58.78	33.82–224.12	0.73–4.65	nd–3.60	nd–3.60	0.63–3.23
		Mean	30.76 ^a^	119.12 ^a^	1.90 ^a^	1.30 ^a^	0.41 ^ab^	1.89 ^a^
		Median	30.93	113.60	1.51	0.90	nd	1.83
100–300	13	Range	19.48–66.58	65.27–319.27	0.65–5.98	nd–2.97	nd–1.80	1.03–4.68
		Mean	31.04^ a^	126.33 ^a^	2.72 ^a^	1.09 ^a^	0.14 ^b^	2.24 ^a^
		Median	29.48	103.35	2.13	1.35	nd	1.68
>300	22	Range	10.28–112.90	16.97–377.17	0.46–19.44	nd–1.05	nd–10.49	0.53–5.48
		Mean	31.37 ^a^	136.13 ^a^	2.71 ^a^	0.24 ^a^	0.73 ^a^	2.68 ^a^
		Median	27.17	109.85	1.13	nd	0.21	2.81

nd: non-detectable. Different letters (superscripts) in the same column indicate the significant difference between the mean contents of different farms (*p* < 0.05) by ^1^ Tukey test and ^2^ Kruskal-Wallis H-test.

**Table 6:** Heavy metals in chicken manure from farms of different herd size (mg/kg dm).

Herd size	No. of farms	Metals	Cu ^2^	Zn ^2^	As ^1^	Cr ^2^	Cd ^2^	Pb ^2^
<2,000	8	Range	19.78–94.73	203.37–394.00	0.75–4.59	nd–65.10	nd–0.60	nd–4.00
		Mean	51.60 ^a^	268.16 ^a^	2.69 ^a^	16.56 ^a^	0.08 ^a^	2.15 ^a^
		Median	43.56	251.97	2.73	4.20	nd	1.81
2,000–20,000	15	Range	1.53–101.93	15.37–367.92	0.68–6.59	nd–2,402.95	nd–6.10	nd–19.93
		Mean	57.22 ^a^	241.74 ^a^	3.33 ^a^	224.79 ^a^	0.67 ^a^	4.89 ^a^
		Median	60.88	252.22	3.17	5.00	nd	2.78
>20,000	11	Range	21.83–487.43	152.17–1,063.32	0.55–10.42	nd–150.1	nd–37.99	0.68–22.10
		Mean	87.14 ^a^	384.15 ^a^	3.79 ^a^	23.71 ^a^	4.05 ^a^	4.44 ^a^
		Median	51.03	348.09	2.34	2.13	nd	2.26

nd: non-detectable. Different letters (superscripts) in the same column indicate the significant difference between the mean contents of different farms (*p* < 0.05) by ^1^ Tukey test and ^2^ Kruskal-Wallis H-test.

**Table 7:** Heavy metals in pig manure from farms of different herd size (mg/kg dm).

Herd size	No. of farms	Metals	Cu ^1^	Zn ^1^	As ^2^	Cr ^2^	Cd ^2^	Pb ^1^
<200	8	Range	266.83–1,337.23	332.47–901.82	1.31–28.38	nd–4.60	nd–203.40	1.38–4.03
		Mean	958.79 ^a^	674.74 ^a^	6.19 ^a^	2.72 ^a^	46.12 ^a^	2.87 ^a^
		Median	1,095.65	737.40	1.91	3.59	0.05	2.84
200–800	9	Range	77.62–890.23	156.47–860.32	1.00–9.51	nd–28.78	nd–0.85	1.18–5.08
		Mean	420.39 ^b^	475.99 ^a^	4.61 ^a^	4.23 ^a^	0.10 ^a^	2.46 ^a^
		Median	516.73	486.17	3.39	1.10	nd	2.28
>800	19	Range	83.43–1,521.43	63.37–1,622.81	0.61–33.48	nd–43.45	nd–111.69	nd–4.53
		Mean	612.23 ^b^	691.56 ^a^	11.75 ^a^	6.56 ^a^	8.38 ^a^	2.37 ^a^
		Median	609.02	721.43	9.94	0.95	0.26	2.53

nd: non-detectable. Different letters (superscripts) in the same column indicate the significant difference between the mean contents of different farms (*p* < 0.05) by ^1^ Tukey test and ^2^ Kruskal-Wallis H-test.

In chicken manures, the contents of Cu, Zn, As, Pb and Cd ranged from 1.53–487.43 mg/kg, 15.37–1,063.32 mg/kg, 0.55–10.42 mg/kg, non-detectable (nd)–22.10 mg/kg and non-detectable (nd)-37.99 mg/kg, respectively. The contents of Cr in chicken manures displayed the widest range, from non-detectable (nd) to 2,402.95 mg/kg. The average contents of Cu, Zn, As, Cr, Cd and Pb in large farms were 87.14, 384.15, 3.79, 23.71, 4.05 and 4.44 mg/kg. There was no significant difference in the contents of heavy metal observed among manures from farms of different herd size. 

In pig manures, the content range of Cu, Zn, As and Cr was 77.62–1,521.43 mg/kg, 63.37–1,622.81 mg/kg, 0.61–33.48 mg/kg and non-detectable (nd)–43.45 mg/kg, respectively. The contents of Cd also clearly featured a large range, from non-detectable (nd) to 111.69 mg/kg. The contents of Pb were typically <5 mg/kg. In large pig farms, the average contents of Zn, As, Cr and Cd in manure were 691.56, 11.75, 6.56 and 8.38 mg/kg, which were generally higher than that in small and middle farms. However, the average content of Cu in manures of small pig farms was significantly higher than that of middle and large farms (*p* < 0.05). The contents of Pb in manures showed comparable values in farms of different herd size. 

## 4. Discussion

### 4.1. Heavy Metals in Animal Feeds

Cu (mostly in the form of copper sulphate) is commonly used as a footbath in milking yards to treat lameness in dairy cattle and as a growth promoter in piggery and poultry units [24]. The pig, cattle, and chicken feeds from animal farms in England and Wales and Jiangsu Province in China contained similar levels of Cu as the feeds tested in our research [14,19]. In terms of different animal feeds, Xiong *et al*. [17] also found the content of Cu in pig feeds was significantly higher than that in cattle and chicken feeds from the farms of Fuxin and Beijing city, which has the same sequence as in this study. The content of Cu in pig feeds varied depending on the herd ages, with higher Cu concentration in weaner and grower-finisher feeds than in sow feeds [16,19], which suggests that farmers tend to use more Cu additives to promote the growth of pigs. 

Zn (mostly in the form of zinc oxide) is added to pig feeds as a “cure-all” for scour whilst Zn is needed by poultry for growth, feather and skeletal development and reproduction, which only requires 50 mg/kg [25]. In England and Wales, the average Zn content in chicken feeds was 2–3 times higher than that required by the birds for healthy development [19]. In this study, it was also found that Zn content in chicken feeds was 1–2 times higher than the amount required. Particularly, it was surprisingly found that there was significantly higher Zn in the feeds from large chicken farms than small farms, which suggested that there would be more Zn application in chicken feeds with intensive farming developing in Northeast China. 

As compounds are generally used to improve the weight gain, the feed efficiency and the pigmentation of animals [15,26]. In this survey, the contents of As in animal feeds also clearly featured a large range from 0.01 to 13.03 mg/kg, which is far beyond the range and the mean values reported in England and Wales [19]. Cang *et al*. [14] reported that in Jiangsu province, the mean As contents of pig, cattle and chicken feeds were 0.09, 0.13 and 0.02 mg/kg, which obviously were lower than those in this survey. However, in Beijing, the mean content of As in pig feeds was 3.2 mg/kg, with the highest value of 37.8mg/kg [15]. Chinese government issued a national standard (GB13078-2001) for the use of As in animal feeds, which limits the content to less than 2 mg/kg [27]. The content of As in about 31.4% pig feed samples was higher than 2 mg/kg, and the proportion of chicken and cattle feeds was 6.7% and 12.8%, respectively. Therefore, more attention should be paid to the excessive addition of As in animal feeds in the Northeast China, especially in pig feeds. 

According to current knowledge, Cd is not added as feed additives for animal growth. Usually, Cd is often present in mineral supplements such as phosphates, Zn sulfate and Zn oxide as an impurity. Thus Cd may enter into the animal production process accompanied with these feed ingredients, which might result in severe dietary contamination. Li *et al*. reported that mean Cd content in pig, cattle and chicken feeds were 2.29, 2.79, and 8.13 mg/kg in Beijing and Fuxin city of China [18]. Besides the high level of Cd in animal feeds, it was reported that Cd accumulation in animal edible offals exceeded the food hygiene criterion of <0.10 mg/kg in Beijing and Jiangsu markets, and it further confirmed the occurrences of Cd in animal production, which might closely relate to the animal feeds [28,29]. Compared with the hygiene standard in GB13078-2001 for animal feeds [27], 20 of 104 feed samples contained Cd was higher than the limit in this survey. Especially, the content of Cd in 31% pig feed samples was higher than 0.5 mg/kg, and the proportion was nearly two times of cattle feeds and three times of chicken feeds. 

### 4.2. Heavy Metals in Animal Manures

The heavy metal contents of animal manures are largely a reflection of their content in the feeds consumed and the efficiency of feed conversion by the animals. Sager [3] reported that mean Cu in pig manure was 282 mg/kg in Austria. Similarly, a survey of England and Wales manures generally found typical content was 350 mg/kg in pig slurry. However, Dong *et al*. [30] found a much higher mean Cu concentration of 1,018 mg/kg in pig manures from the pig farms in Hangzhou, a large city in the Yangtze River Delta, China. Li *et al*. [16] reported that average Cu in manures from Beijing city was 887 mg/kg in grower-finisher pig manures, which was higher than those from middle and large pig farms but lower than that from small farms in this study. In the Northeast China, small farms are widely distributed in rural areas, where heavy metal additives were abused in feeds due to the insufficient supervision of governments and less professional knowledge. As a result, excessive application of Cu additives to feeds result in high Cu residues in manures from small pig farms. 

The application of As additives in animal feeds resulted in various As residues in manures [15,26]. In England and Wales, the average contents of As in pig, layer and cattle manure were 1.68, 1.46 and 0.44 mg/kg [19]. Sager *et al*. [3] reported that the average contents of As in pig and cattle manure was <1.0 mg/kg in Austria. Both these results are significantly lower than that in Northeast China in this study. However, Jackson *et al*. [26] reported that the mean content of As in chicken manure was 15.7 mg/kg in Alabama, Georgia, and South Carolina, USA. Yao *et al*. [20] and Li *et al*. [15] also found that the much higher content of As in pig manure was 89.3 mg/kg in Guangdong province and 19.7 mg/kg in Beijing, which were developed areas of livestock husbandry. Li *et al*. [15] further suggested arsenic content in pig manure was greatly enhanced along the increasing of arsenic in pig feed. 

In terms to the average content, pig and chicken manures contained higher Cd than cattle manure [18], which is consistent with the current study. According to the report of Sager [3], the mean content of Cd in chicken manure and pig manure in Austria <0.5 mg/kg, which was comparable the survey of chicken manures in USA [26]. Both in this study and the surveys of chicken and pig manures in Beijing and Fuxin city, the contents in China were much higher than these results. However, the median content of pig, chicken and cattle manures is <0.3 mg/kg. The average contents of Cd in pig, chicken and cattle manures were very high because of some extreme high values found in this survey, such as 203.40 mg/kg in a pig manure sample. Extremely high values of Cd in pig manures have been reported in other areas of China, e.g., 129.8 mg/kg in Beijing and 120.1 mg/kg in Jilin province [17,31]. Actually, the limit of Cd content for the land application of animal manure should follow the requirements of GB8172-87 (Cd < 3.0 mg/kg, dm) in China [32], and in this study, 10 of 118 manure samples exceeded the limit. In detail, the content of Cd in 11.1% pig manure sample, 11.7% chicken manure sample and 4.3% cattle manure sample was higher than the limit. As shown in our previous study, there were significant correlations between Cd concentrations in the feeds and manures of pig, dairy cow and chicken [17]. Therefore, the addition of Cd to animal feeds, especially pig and chicken feeds, results in high Cd residues in manures, which would pose high Cd pollution risk to farmlands in the Northeast China. 

## 5. Conclusions

Feed additives containing heavy metals were widely applied in intensive animal production industry in Northeast China, and the abuse of additives with heavy metals occurred in both large farms and small farms. Generally, the contents of heavy metals are higher in pig feeds than in chicken and cattle feeds, and consequently, pig manure could pose a higher risk of heavy metal pollution to farmlands than chicken and cattle manures. From the aspect of environmental protection, animal feed additives in Northeast China should be controlled based on the relevant legal limits in China. Furthermore, we shall estimate the load of heavy metals on farmlands caused by animal manure and the loss to rivers in future work. 
